# White-Opaque Switching in Natural *MTL*a/α Isolates of *Candida albicans*: Evolutionary Implications for Roles in Host Adaptation, Pathogenesis, and Sex

**DOI:** 10.1371/journal.pbio.1001525

**Published:** 2013-03-26

**Authors:** Jing Xie, Li Tao, Clarissa J. Nobile, Yaojun Tong, Guobo Guan, Yuan Sun, Chengjun Cao, Aaron D. Hernday, Alexander D. Johnson, Lixin Zhang, Feng-Yan Bai, Guanghua Huang

**Affiliations:** 1 State Key Laboratory of Mycology, Institute of Microbiology, Chinese Academy of Sciences, Beijing, China; 2 University of Chinese Academy of Sciences, Beijing, China; 3 Department of Microbiology and Immunology, University of California, San Francisco, San Francisco, California, United States of America; 4 Chinese Academy of Sciences Key Laboratory of Pathogenic Microbiology and Immunology, Institute of Microbiology, Chinese Academy of Sciences, Beijing, China; Duke University Medical Center, United States of America

## Abstract

Phenotypic transitions play critical roles in host adaptation, virulence, and sexual reproduction in pathogenic fungi. A minority of natural isolates of *Candida albicans*, which are homozygous at the mating type locus (*MTL*, **a**/**a** or α/α), are known to be able to switch between two distinct cell types: white and opaque. It is puzzling that white-opaque switching has never been observed in the majority of natural *C. albicans* strains that have heterozygous *MTL* genotypes (**a**/α), given that they contain all of the opaque-specific genes essential for switching. Here we report the discovery of white-opaque switching in a number of natural **a**/α strains of *C. albicans* under a condition mimicking aspects of the host environment. The optimal condition for white-to-opaque switching in **a**/α strains of *C. albicans* is to use N-acetylglucosamine (GlcNAc) as the sole carbon source and to incubate the cells in 5% CO_2_. Although the induction of white-to-opaque switching in **a**/α strains of *C. albicans* is not as robust as in *MTL* homozygotes in response to GlcNAc and CO_2_, opaque cells of **a**/α strains exhibit similar features of cellular and colony morphology to their *MTL* homozygous counterparts. Like *MTL* homozygotes, white and opaque cells of **a**/α strains differ in their behavior in different mouse infection models. We have further demonstrated that the transcriptional regulators Rfg1, Brg1, and Efg1 are involved in the regulation of white-to-opaque switching in **a**/α strains. We propose that the integration of multiple environmental cues and the activation and inactivation of a set of transcriptional regulators controls the expression of the master switching regulator *WOR1*, which determines the final fate of the cell type in *C. albicans*. Our discovery of white-opaque switching in the majority of natural **a**/α strains of *C. albicans* emphasizes its widespread nature and importance in host adaptation, pathogenesis, and parasexual reproduction.

## Abstract 2

All *Mating Type Locus* strain types of *Candida albicans* show white-opaque switching competency, not just MTL homozygotes, which allows them to adapt better to environmental changes.

## Author Summary

Phenotypic transitions enable fungal pathogens to better adapt to their ever-changing environments. Approximately 10% of natural *Candida albicans* strains, which are homozygous at the mating type locus (*MTL*, **a**/**a** and α/α), can switch between two distinguishable morphological forms: white and opaque. The two cell types differ in a number of biological aspects including virulence, susceptibility to host immune attacks, and mating competency. Here, we demonstrate that white-opaque switching competency is not restricted to the *MTL* homozygous strains, but is a general characteristic of all *MTL* strain types of *C. albicans* (**a**/**a**, α/α, and **a**/α). Two host environmental cues, N-acetylglucosamine and CO_2_, promote white-to-opaque switching and stabilize the opaque phenotype. Thus, although switching is normally blocked in **a**/α cells, this block can be overcome through specific environmental changes. We further show that three transcriptional regulators (Rfg1, Brg1, and Efg1) help to regulate white-opaque switching in *MTL* heterozygotes of *C. albicans*. This study generalizes white-opaque switching to strains with all mating-type configurations and emphasizes its importance in host adaptation, pathogenesis, and parasexual reproduction.

## Introduction

Phenotypic plasticity is critical for microorganisms to survive under fluctuating environments. For fungal pathogens, phenotypic switching is a common strategy to rapidly adapt to different host niches and facilitate colonization and infection [1]. A specific phenotype can also confer the fungus a growth advantage over competing microorganisms in a specific environment or host niche. *Candida albicans*, the major causative agent of fungal infections in humans, can switch between two different visible cell types: white and opaque [2]. The two cell types differ in a number of biological aspects including morphology, virulence, and mating competence [3]–[5]. White cells are small and round and form “white,” dome-shaped colonies on solid media, while opaque cells are large and elongated and form darker and flatter colonies [6]. White cells are more virulent than opaque cells in systemic infections, whereas opaque cells appear more suited to cutaneous colonization [7],[8]. Opaque cells possess pimples on the cell wall and exhibit unique antigenicity, which may help the pathogen in evading the host immune system [3]–[5]. Moreover, opaque cells are significantly less susceptible to phagocytosis by cells of the fly and mouse innate immune systems than white cells [9]. Perhaps the best studied feature of opaque cells is their mating competency. Opaque cells mate ∼10^6^ times more efficiently than white cells [10]. It has recently been shown that *Candida tropicalis*, another important human fungal pathogen, can also undergo white-opaque switching and parasexual mating [11],[12].

Despite the importance of white-opaque switching in host adaptation, pathogenesis, and parasexual reproduction in *C. albicans*, only a minority (<10%) of natural strains have been reported to undergo white-opaque switching in vitro [13]. It has been shown that the mating-type locus homeodomain proteins (MTL**a**1/α2) inhibit white-opaque switching via controlling the expression of the master regulator *WOR1* [14]–[16]. Consistent with this, the minority of natural isolates capable of white-opaque switching in vitro are homozygous at the *MTL* locus; this relieves the block of the mating locus proteins. The majority (>90%) of *C. albicans MTL* **a**/α isolates in nature were thought to be incapable of white-opaque switching unless they underwent homozygosis of the mating type locus [13]. These ideas raised a fundamental question. Given the importance of white-opaque switching in host adaptation and pathogenesis, why do the majority of **a**/α natural isolates of *C. albicans* not undergo white-opaque switching unless they undergo a genetic rearrangement?

In this study, we provide reasonable answers to this basic question. We show that naturally occurring **a**/α isolates of *C. albicans* can indeed undergo white-opaque switching under a specialized set of environmental conditions. Previous studies typically used glucose as the sole carbon source and grew cells in ambient CO_2_. We show here that **a**/α strains can undergo white-opaque switching in 5% CO_2_ when N-acetylglucosamine (GlcNAc) is used as the sole carbon source. GlcNAc and CO_2_, primarily produced by bacterial commensals, are abundant in the gut and have synergistic effects on the induction of the opaque cell phenotype in *C. albicans* [17]. Therefore, this culture condition likely mimics certain aspects of host niches, such as those in the gut. Opaque cells of **a**/α strains exhibit similar phenotypes of typical *MTL* homozygous opaque cells, except they lack the ability to mate. Further experiments demonstrate that three transcription factors Rfg1, Brg1, and Efg1 are involved in the regulation of white-opaque switching in **a**/α *C. albicans* strains. This study indicates that there is an alternative gene circuit, which can bypass the **a**1/α2 block to switching, and promote white-opaque switching in *C. albicans* under certain environmental conditions that are reminiscent of niches of the host. We propose that white-opaque switching is not limited to the minority of *MTL* homozygotes, but rather is a general characteristic of natural *C. albicans* strains.

## Results

### Demonstration of White-Opaque Switching in Natural *MTL*a/α Strains of *C. albicans*

There are three *MTL* types of natural *C. albicans* isolates (**a**/**a**, α/α, and **a**/α). Under normal conditions, *MTL* heterozygotes (**a**/α) are blocked for switching and “locked” in the white phase in vitro [10],[13]. Since **a**/α strains are more competitive than their **a**/**a** or α/α derivatives (at least in some in vivo assays) and carry the entire set of opaque-specific genes essential for switching [18], we suspected that the **a**/α isolates of *C. albicans* could also undergo white-opaque switching in their natural niches. We also reasoned that routine laboratory media and culture conditions were totally different from conditions in natural niches and might not be conducive for the transition in **a**/α strains of *C. albicans*. To test our hypothesis, we took advantage of the synergistic effects of two host environmental cues, GlcNAc and CO_2_, on the induction of the opaque cell phenotype [17]. We grew 94 natural isolates of *C. albicans* on Lee's GlcNAc medium in 5% CO_2_. We found that 34 strains (36%) formed opaque colonies under this condition. We then examined the *MTL* genotype of all 94 tested strains. Of them, 92 were **a**/α, one was **a**/**a**, and one was α/α. The two *MTL* homozygotes (one **a**/**a** and one α/α) were identified as switching to opaque, along with the 32 **a**/α strains in the switchable strain list (Table S1).

**Figure 1:**
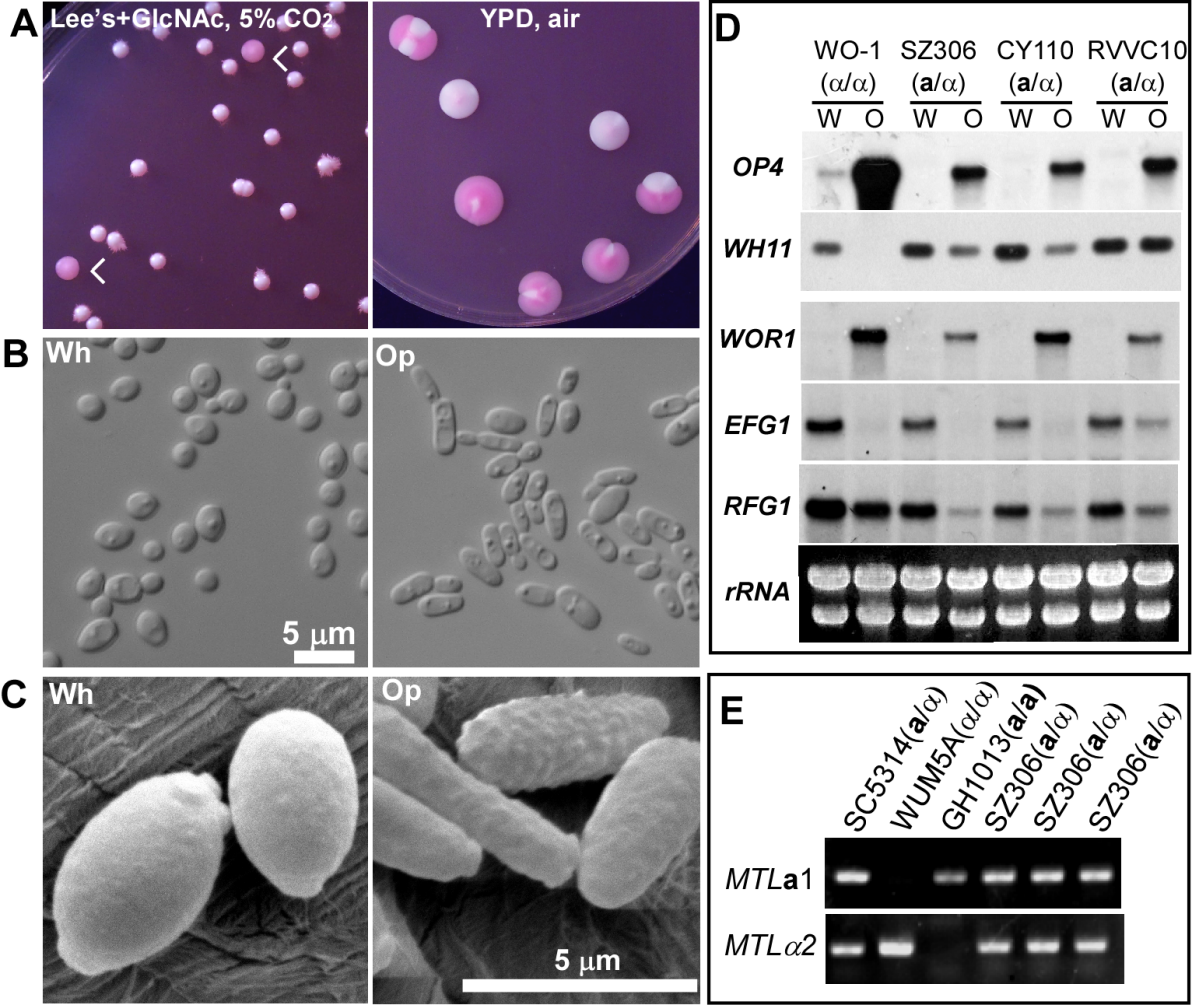
White-opaque switching in a natural *MTL*a/α strain of *C. albicans*. (A) Colony images of SZ306, a clinical *MTL* **a**/α isolate, grown on Lee's GlcNAc medium in 5% CO_2_ (left) for 7 days and on YPD medium in air for 10 days at 25°C. The dye phloxine B, which exclusively stains opaque colonies red, was added to the media. White arrows indicated the opaque colonies on Lee's GlcNAc medium. Pink colonies were opaque on YPD medium. (B) Cellular morphology of white and opaque cells of SZ306 (**a**/α). Cells were collected from YPD cultures and imaged. Scale bar, 5 µm. (C) SEM images of white and opaque cells of SZ306 (**a**/α). Cells were collected from YPD cultures and imaged. Scale bar, 5 µm. (D) Northern blot of white and opaque enriched genes in three independent natural **a**/α isolates and the reference strain WO-1. (E) PCR of *MTL* **a**1 and α2 genes in the white-opaque switchable SZ306 (**a**/α). Primers used for PCR are listed in Table S6. The previously characterized strains (SC5314, WUM5A, and GH1013) served as *MTL* **a**/α, α/α, and **a**/**a** controls, respectively. Three independent opaque colonies of SZ306 were tested.

An example of an **a**/α clinical strain that could undergo white-opaque switching (SZ306) is shown in Figure 1. We noticed that SZ306 could also form opaque colonies on rich medium (YPD) when cultured for an extended time period (Figure 1A); some other **a**/α strains also exhibited this behavior. The white and opaque cells of **a**/α strains were similar to their counterparts of *MTL* homozygotes in the size and shape of cells (Figure 1B): white cells of **a**/α strains were small and round with no pimples on their cell wall surface, while opaque cells were elongated and possessed obvious opaque-specific pimples (Figure 1C). Northern blot analysis demonstrated that two opaque-enriched genes, *OP4* and the master regulator *WOR1*, were expressed in opaque cells of **a**/α strains but not in white cells (Figure 1D). Conversely, the expression levels of the white-enriched genes *WH11*, *EFG1*, and *RFG1* were significantly higher in white cells than in opaque cells of **a**/α strains. These results suggest that opaque cells of **a**/α strains exhibit similar characteristics of colony and cellular morphology and gene expression profile to the opaque cells of *MTL* homozygotes.

To exclude the possibility of homozygosis of **a**/α cells during growth, we re-plated several opaque colonies of each switchable **a**/α strain onto Lee's GlcNAc medium and incubated them in ambient CO_2_ for 5 days. Three single opaque colonies of each re-plated culture were examined for the *MTL* configuration, and we verified that all remained heterozygous at the *MTL* locus. An example of this analysis is given in Figure 1E. These results demonstrate that *C. albicans* **a**/α isolates can indeed undergo white-opaque switching.

### *MTL*a/α White-Opaque Switchable Strains of *C. albicans* Are Genetically Diverse

Additional examples of white-opaque switching in natural **a**/α strains of *C. albicans* are shown in Figure S1 and Table S1. The white colonies of different **a**/α strains showed variability in their abilities to filament on Lee's GlcNAc medium in 5% CO_2_ at 25°C, indicating that the white-opaque switchable strains are genetically diverse and probably not derived from a single strain with a specific genetic background. To characterize the genetic background of these natural strains, we sequenced their CAI microsatellite loci by using a reported assay [19]. As shown in Table S1 (Column D), these strains exhibited several distinct patterns of the CAI genotype, demonstrating their genetic diversity.

The strains listed in Table S1 were all isolated in China. To exclude the possibility of geographical specificity, we tested the white-to-opaque switching ability in 29 clinical strains of *C. albicans* isolated from different countries. These strains, which were demonstrated incapable of switching on glucose-containing media, were all originally heterozygous at the *MTL* locus (**a**/α) and belonged to five different genetic clades [13]. We found that 15 of them (52%) underwent the white-to-opaque transition on Lee's GlcNAc medium in 5% CO_2_ at 25°C (Table S2). Two opaque colonies of each switchable strain were examined for the *MTL* configurations. Twelve of the 15 strains were **a**/α heterozygotes, two (P75010, P22095) α/α, and one **a**/**a** (P78042, perhaps due to spontaneous loss of the *MTL*α locus, Pujol and Soll, unpublished data). These results further indicate that the white-opaque switchable **a**/α strains of *C. albicans* are genetically and geographically diverse.

### Opaque Cells of *C. albicans MTL*a/α Strains Are Mating-Incompetent

**Figure 2:**
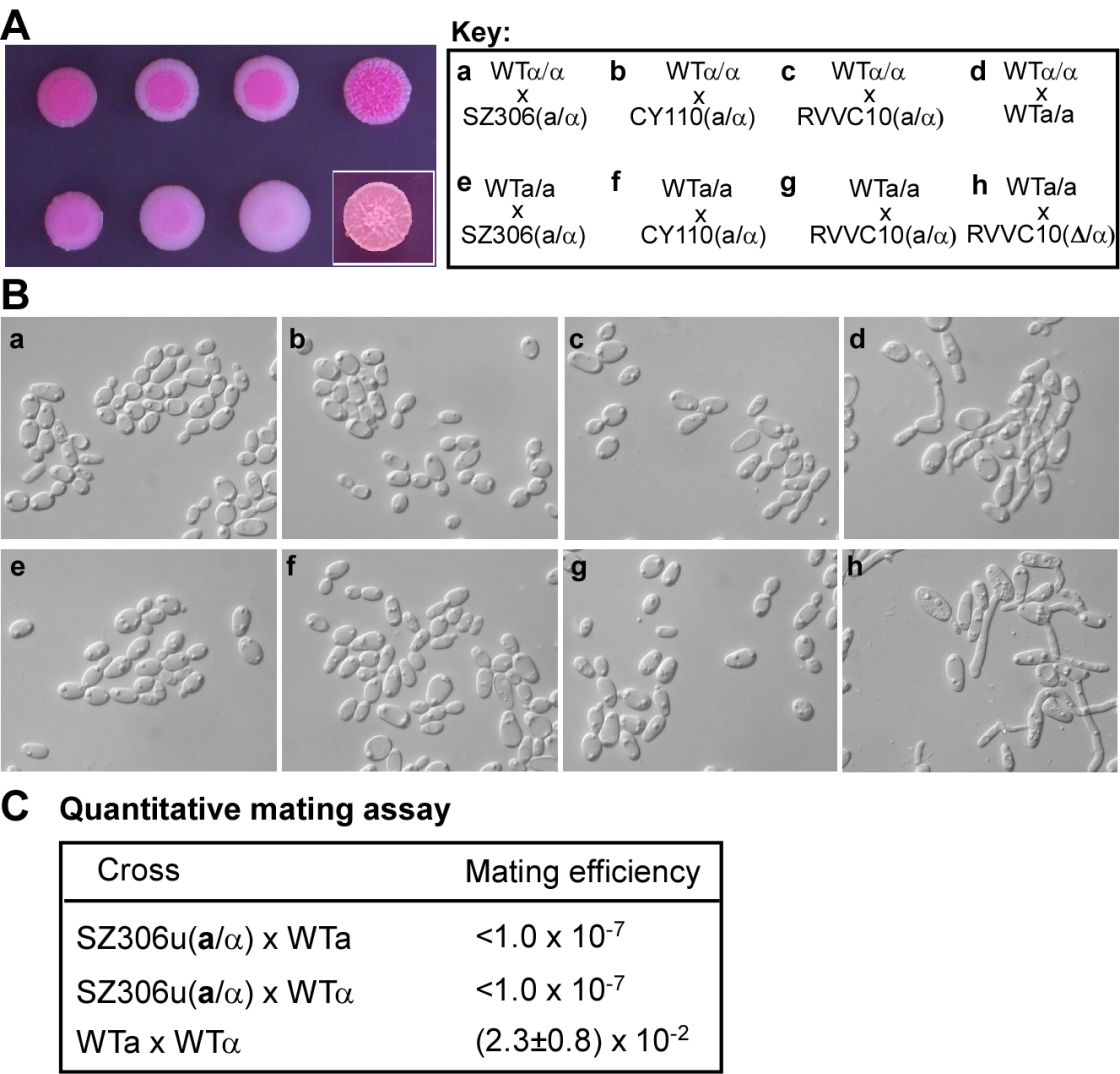
Opaque cells of *C. albicans MTL*a/α strains cannot mate with a/a or α/α opaque cells. 10^6^ opaque cells of each mating partner were mixed and spotted onto Lee's GlcNAc agar and incubated at 25°C for 4 days. (A) Patches of **a**/α cells and **a**/**a** or α/α mixture on Lee's GlcNAc medium at 25°C. Three natural **a**/α isolates were tested. WT**a**/**a** (GH1012) and α/α (WO-1) mixture served as a positive control of mating assay. Wrinkle surface indicated the formation of long mating conjugation tubes. (B) Mating response of corresponding patches in panel A. Cell fusion and mating conjugation tubes were only observed in the crosses of (d) and (h). (C) Quantitative mating assay. SZ306u (*MTL* **a**/α, *ura3-*), WT**a** (SN152a, *MTL* **a**/Δ, *arg4-his1-leu2-*), WTα (GH1349, a derivative of WUM5A, *MTL*α/α, *agr4-*, used for the SZ306u × WTα cross), and WTα (WUM5A, *MTL*α/α, *ura3-*, used for the control WTα × WT**a** cross). Mating efficiency = average ± SD. “<” indicated no prototropic colonies were observed. Opaque cells were used for all the mating crosses.

White-opaque switching and mating are two coupled biological processes that are both controlled by the MTL**a1**/α2 complex in *C. albicans* [10]. One possibility that could explain how **a**/α isolates could undergo white-opaque switching is that the **a**1/α2 complex might not function properly; thus, cells could behave as though they were **a** or α cells. Although our DNA sequencing analysis showed that the *MTL* locus of the switchable **a**/α isolates were normal and with no obvious defects, the expression of *MTL* **a**1 or *MTL*α2 could, in principle, be defective. To exclude this possibility, we performed a mating experiment with opaque cells from three independent **a**/α strains. As shown in Figure 2A, these cells showed no mating response, whereas **a**/**a** and α/α opaque cell controls mated normally (Figure 2Ba, b, c, e, f, and g). Quantitative mating assay demonstrated that the mating efficiencies of the *MTL* **a**/α x *MTL* **a** or α cells crosses were undetectable (<1×10^−7^). The mating efficiency of the *MTL* **a**/Δ x α/α cross-control was (2.3±0.8)×10^−2^, at least 1×10^5^ times higher than that of the *MTL* **a**/α crosses (Figure 2C). These results demonstrate that **a**/α opaque cells cannot mate with either **a**/**a** or α/α opaque cells, suggesting that the white-opaque switchable **a**/α strains are mating-incompetent. However, once the opaque cells of these **a**/α strains were converted to **a**/Δ or Δ/α strains by deletion of one allele of the *MTL* locus, they acquired mating competence and mated as efficiently as the WT **a**/**a** or α/α controls (Figure 2Ah). We conclude from these experiments that the **a**1/α2 complex is functional in the regulation of mating, and that the white-opaque switching in these strains is not due to the inactivation of **a**1 or α2 proteins.

### Induction of White-to-Opaque Switching by GlcNAc and CO_2_ in *C. albicans MTL*a/α Strains

**Figure 3:**
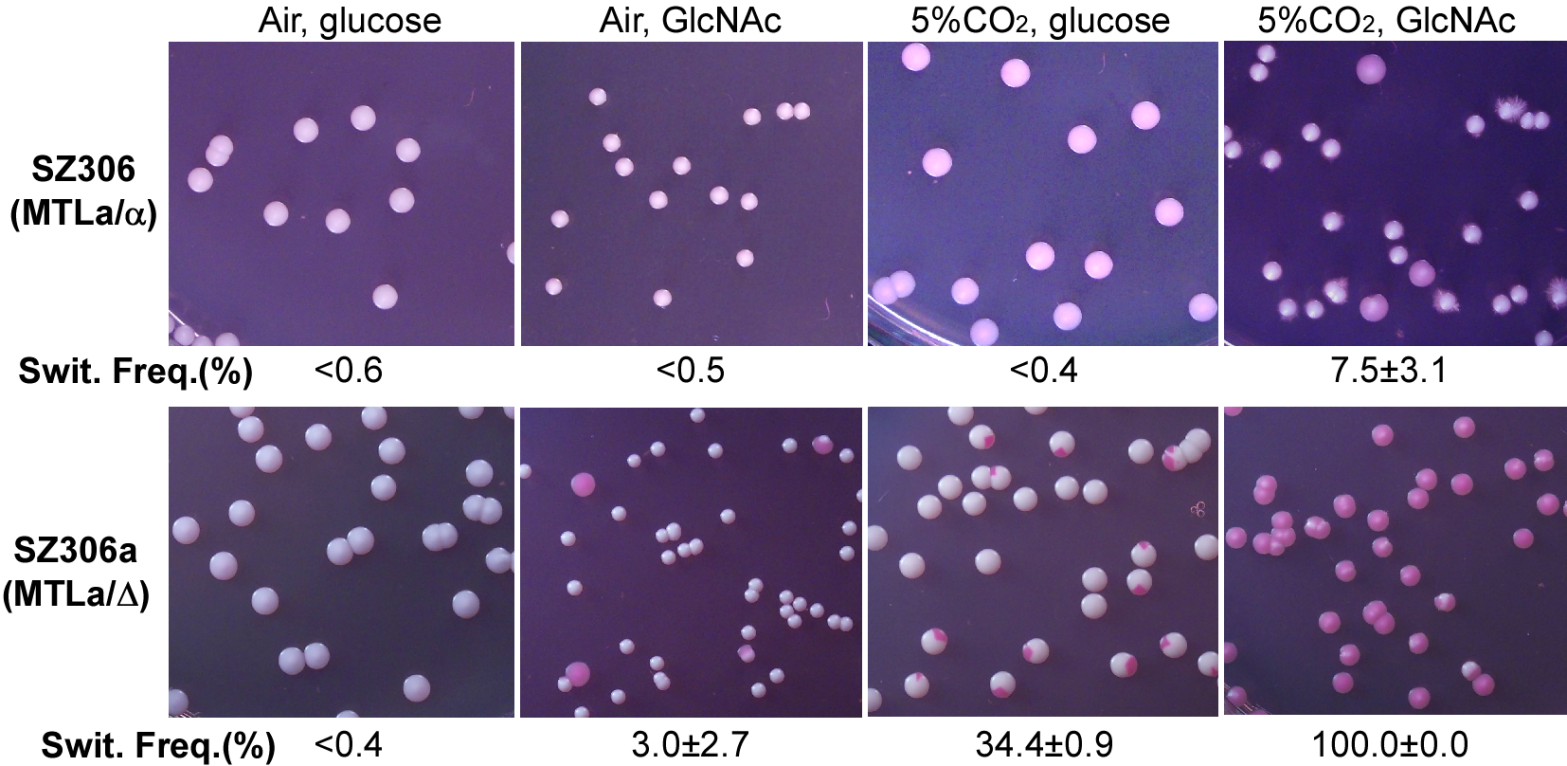
*MTL*a/Δ derivatives are more sensitive to GlcNAc and CO_2_ than natural a/α strains. White-to-opaque switching in SZ306 (**a**/α) and SZ306a (**a**/Δ) were tested under four conditions indicated. Lee's glucose or GlcNAc medium was used for cell growth. Homogeneous white cells from Lee's glucose plates were plated. The total colony number examined for each strain was 350 to 500. Switching frequencies (% of average ± standard deviation, SD) are shown below the images. “<” indicated no opaque or opaque-sectored colonies were observed.

As described in the introduction, GlcNAc and CO_2_ are two potent inducers of white-to-opaque switching and are believed to be characteristic of host niches such as the gastrointestinal (GI) tract [17]. As shown in Figure 3, the frequencies of white-to-opaque switching in the **a**/α strain SZ306 was extremely low on Lee's glucose (<0.6%) or GlcNAc (0.5%) medium in ambient CO_2_. CO_2_ alone also had little effect on the induction of opaque phenotype on Lee's glucose medium in this **a**/α strain (switching frequency<0.4%). However, the switching frequency of white-to-opaque in SZ306 was increased to 7.5±3.1% when cultured on Lee's GlcNAc medium in 5% CO_2_, indicating that GlcNAc and CO_2_ had a synergistic effect on the induction of the opaque cell phenotype. To compare the switching features of **a**/α strains and *MTL* homozygous “**a**” or “α” strains, we converted SZ306 (**a**/α) to an *MTL* **a**/Δ strain, namely SZ306a, and RVVC10 (**a**/α) to an *MTL*Δ/α strain, namely RVVC10α, by deletion of one allele of the *MTL* locus. As shown in Figure 3, although the frequency of white-to-opaque switching in SZ306a was only 0.4% on Lee's glucose medium in ambient CO_2_, GlcNAc, or 5% CO_2_ alone increased the switching frequencies to 3.0±2.7% and 34.4±0.9%, respectively. Notably, SZ306a underwent a mass conversion (switching frequency = 100%) on Lee's GlcNAc medium in 5% CO_2_, consistent with our previous study of the synergistic effect of GlcNAc and CO_2_ on white-to-opaque switching in *MTL* homozygotes [17]. As in SZ306 and SZ306a, GlcNAc and CO_2_ had a similar effect on the induction of the opaque cell phenotype in RVVC10 and its derivative, RVVC10α (unpublished data). These results indicate that **a**/α strains are less sensitive than their “**a**/Δ” or “Δ/α” derivatives to GlcNAc and CO_2_, but that white-to-opaque switching is stimulated by GlcNAc and CO_2_ in all three *MTL* configurations.

GlcNAc and CO_2_ can also stabilize the opaque phenotype in *MTL* homozygotes of *C. albicans*. We next tested whether this was also the case in heterozygous **a**/α strains. As shown in Figure S2 and Table S3, the opaque phenotype of **a**/α strains was extremely unstable in Lee's glucose medium when cultured in ambient CO_2_ at 25°C (switching frequency to white was 100%). The switching frequencies were 38.6±7.7, 34.6±5.3, and 18.7±7.1 on Lee's GlcNAc medium in ambient CO_2_, on Lee's glucose in 5% CO_2_, and on Lee's GlcNAc in 5% CO_2_, respectively (Figure S2 and Table S3). These results suggest that GlcNAc and CO_2_ stabilize the opaque phenotype of **a**/α strains. For the “**a**/Δ” and “Δ/α” strains, SZ306a and RVVC10α, the opaque phenotype was very stable on both Lee's glucose and GlcNAc, irrespective of whether the cells were cultured in air or 5% CO_2_. Under the four conditions tested (Lee's glucose in air, Lee's GlcNAc in air, Lee's glucose in 5% CO_2_, and Lee's GlcNAc in 5% CO_2_), the opaque-to-white switching frequencies of SZ306a and RVVC10α were all less than 1% (Figure S2 and Table S3). We sequenced the *WOR1* promoter of several switchable **a**/α strains and found what is believed to be the major **a**1/α2 cis-regulatory sequence site was intact. These results indicate that although the **a**1/α2 complex does not provide an absolute block to white-to-opaque switching in these **a**/α strains, it reduces switching to favor white cells, likely by turning down (but not off) the expression of *WOR1*.

### *C. albicans MTL*a/α Strains Undergo White-to-Opaque Switching at the Host Physiological Temperature

**Figure 4:**
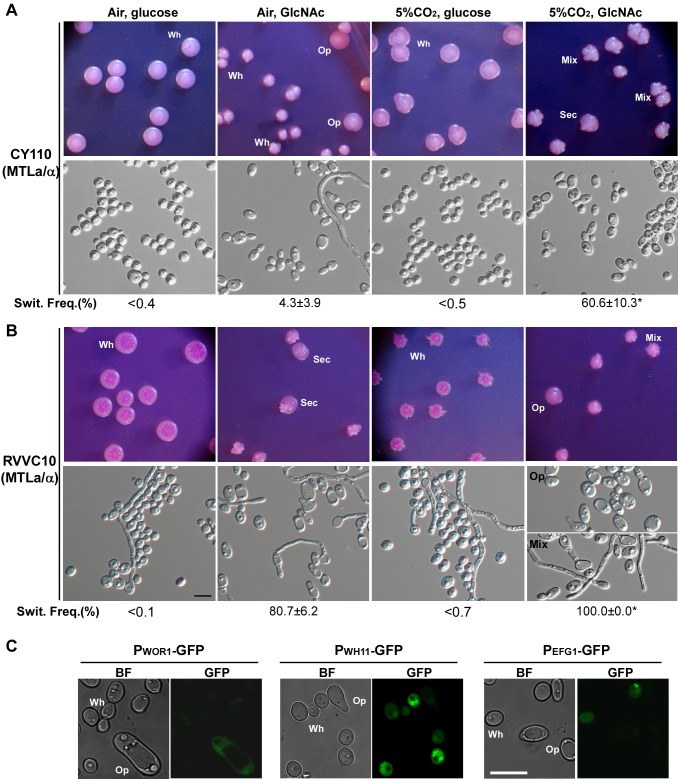
White-to-opaque switching in natural *MTL*a/α strains of *C. albicans* at 37°C. White cells of two *MTL* **a**/α strains (CY110 and RVVC10) were plated onto Lee's glucose and Lee's GlcNAc medium plates and cultured in air or 5% CO_2_ at 37°C for 3 to 4 days. At this temperature, white cell colonies containing filamentous swollen cells could be stained red. Cellular morphologies were examined to confirm the cellular phenotypes. *, the switching frequencies (% of average ± SD) of these samples represented the percentages of colonies containing opaque cells, including opaque, opaque-sectored, and white-opaque mixed colonies. Swit. Freq., switching frequency. Wh, white cells/colonies. Op, opaque cells/colonies. Scale bar, 10 µm. (A) White-to-opaque switching of CY110 at 37°C. (B) White-to-opaque switching of RVVC10 at 37°C. (C) Expression of GFP in the reporter strains of *WOR1/WOR1::WOR1p-GFP*, *WH11/WH11::WH11p-GFP*, and *EFG1/EFG1::EFG1p-GFP*. The parent strain of these strains was CY110 (*MTL* **a**/α). Opaque-sectored colonies grown on Lee's GlcNAc medium plates (without phloxine B) were microscopically examined. BF, bright field.

Since the physiological temperature of human hosts is 37°C, we therefore examined whether *MTL* **a**/α strains can undergo white-to-opaque switching under this temperature. White cells of CY110 and RVVC10 (two *MTL* **a**/α strains) were plated onto Lee's glucose and Lee's GlcNAc medium plates and cultured at 37°C for 3 to 4 days. The cells of both strains were locked in white phase on Lee's glucose medium in air or in 5% CO_2_, whereas they formed opaque, opaque-sectored, or mixed colonies on Lee's GlcNAc medium (Figure 4A and 4B). The switching frequencies of CY110 and RVVC10 on Lee's GlcNAc medium in 5% CO_2_ were as high as 60.6±10.3% and 100% (mass conversion), respectively. The cellular morphologies demonstrated that opaque or mixed colonies contained typical opaque cells (Figure 4A and 4B). *WOR1* is an opaque phase-specific gene, while *WH11* and *EFG1* are white phase-specific genes [5]. To further verify their cell identities, we constructed *WOR1*, *WH11*, and *EFG1* promoters-controlled GFP reporter strains in the *MTL* **a**/α strain CY110. As shown in Figure 4C, GFP fluorescence was only observed in opaque cells of the *WOR1/WOR1::WOR1p-GFP* strain, but not in opaque cells of the *EFG1/EFG1::EFG1p-GFP* and *WH11/WH11::WH11p-GFP* strains. As expected, GFP fluorescence was observed in white cells of the *EFG1/EFG1::EFG1p-GFP* and *WH11/WH11::WH11p-GFP* strains. These results indicated that the opaque cells formed at 37°C were genetically opaque.

We next tested the stability of opaque cells of *MTL* **a**/α strains under host physiological temperature. Opaque cells of three **a**/α strains (SZ306, RVVC10, and CY110) were plated onto Lee's glucose and Lee's GlcNAc medium and incubated in air at 37°C for 3 days. On Lee's glucose medium, opaque cells underwent a mass conversion to the white cell phase (switching frequency = 100%). On Lee's GlcNAc medium, most colonies (>95%) remained in the opaque phase. The cellular morphology of representative colonies is shown in Figure S3, indicating that GlcNAc can stabilize the opaque phenotype of **a**/α strains at 37°C.

### White and Opaque Cells of *C. albicans MTL*a/α Strains Differ in Fungal Burden in Systemic and Cutaneous Infections

**Figure 5:**
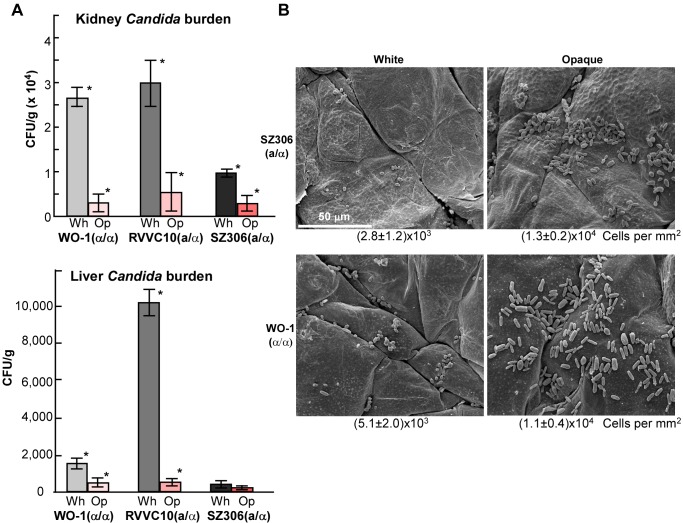
White and opaque cells differ in fungal burden in systemic and cutaneous infections. (A) Fungal burdens of the kidneys and livers of systemically infected mice are shown. Each male mouse was intravenously injected with 200 µl 1× PBS containing 2×10^6^ cells via the tail vein. Three to four mice per strain were used for the injections. Mice were sacrificed on the 3^rd^ day postinfection. CFUs, colony-forming units. White and opaque cells of an *MTL* **a**/α strains, RVVC10 and SZ306, were tested. The *MTL*α/α strain, WO-1, served as the control. Error bars stand for standard deviation (SD). * indicated significant difference (op. versus wh., Student's *t* test *p* value<0.05). (B) SEM images of skin colonization in a newborn mouse model. White and opaque cells of an *MTL* **a**/α strain, SZ306, were tested. The *MTL*α/α strain, WO-1, served as the control. The number of colonized cells (average ± SD, cells per mm^2^) is shown below the images. Five randomly selected fields of view were counted. The number of opaque cells that colonized the skin was significantly higher than that of white cells (Student's *t* test *p* value<0.002).

In *MTL* homozygous strains of *C. albicans*, white and opaque cells show differences in their behaviors in systemic and skin infection models [7],[8]. White cells are more virulent in systemic mouse model than opaque cells, while opaque cells are better at cutaneous infections. We then tested whether white and opaque cells of *C. albicans MTL* **a**/α strains also differed in virulence in different infection models. As shown in Figure 5A, in a systemic mouse infection system, burdens of opaque cells in the liver were notably less than those of white cells of RVVC10 and SZ306 (Student's *t* test *p* value<0.05), suggesting opaque cells of *C. albicans* **a**/α strains proliferated or colonized less well than their white cell counterparts. This was also the case for colonization of the kidney for RVVC10, although the difference of fungal cell burden between white and opaque cells of SZ306 was not significant. This result is consistent with previous studies; the fungal burdens of opaque cells of the *MTL* homozygous reference strain WO-1 in both the kidney and liver were less than those of white cells of WO-1 [7], and the fungal burdens of opaque cells of the *MTL* homozygous reference strain WO-1 in both the kidney and liver were less than those of white cells of WO-1. To test whether opaque cells of **a**/α strains were better at cutaneous infections, newborn mice were used and the fungal colonization of the skin was assessed by scanning electron microscopy as described previously [8]. Compared to white cells, opaque cells of both SZ306 and the reference strain WO-1 showed increased colonization in a cutaneous mouse model (Figure 5B). The number of opaque cells that colonized the skin was significantly higher than that of white cells (Student's *t* test *p* value<0.002) (Figure 5B). These results indicate that the different behaviors documented for white and opaque cells in the systemic and cutaneous mouse models also apply to white and opaque cells of the **a**/α strains described here.

### Global Transcriptional Profiles of White and Opaque Cells of the *MTL*a/α Strain CY110

To characterize the genome-wide transcriptional profiles of white and opaque cells of the *MTL* **a**/α strains, we performed RNA-Seq analysis of CY110, a clinical isolate of *MTL* **a**/α genotype. As shown in Table S4 (Sheet 1), the expression levels of 1,631 genes demonstrated a greater than twofold change in white and opaque cells. As expected, previously characterized white cell–enriched genes, such as *WH11* and *EFG1*, were up-regulated in white cells, while opaque cell–enriched genes, such as *WOR1* and *OP4*, were strongly up-regulated in opaque cells. A total of 838 genes demonstrated a greater than 3-fold change in our RNA-Seq analysis. Of them, 459 were previously reported as white (205) or opaque (254) cell–enriched genes [20],[21], and 379 were only found in our analysis, which could be *MTL* genotype-dependent phase-specific genes. As shown in Table 1, of the highly differentially expressed genes, the ratio of potential *MTL* genotype-dependent genes remarkably decreased, suggesting that highly differentially expressed genes are less *MTL* genotype-dependent. Interestingly, many cell wall protein and biofilm-induced genes were among the *MTL* **a**/α-specific genes (Table S4, sheet 3). Of note, the *MTL* **a**/α-specific genes may contain a proportion of genes specific to the strain background, especially for those with lower fold-change of expression levels.

**Table 1:** RNA-Seq analysis of the differentially expressed ORFs in white and opaque cells of CY110.

	3-Fold Changes		8-Fold Changes		16-Fold Changes	
Differential Expressed ORFs (*n*)	Wh(up)	Op (up)	Wh (up)	Op (up)	Wh (up)	Op (up)
Total	389	449	115	176	69	114
Potential a/α strain specific ORFs	184 (47.3%)	195 (43.4%)	17 (14.8%)	32 (18.2%)	10 (14.5%)	18 (15.8%)
*MTL*-independent ORFs	205 (52.7%)	254 (56.6%)	98 (85.2%)	144 (81.8%)	59 (85.5%)	96 (84.2%)

Wh(up), up-regulated in white cells; Op(up), up-regulated in opaque cells. Total, total number of differential expressed ORFs in white or opaque cells of CY110; potential **a**/α strain specific ORFs, number of differential expressed ORFs only found in white or opaque cells of CY110; *MTL*-independent ORFs, number of differential expressed ORFs found in white or opaque cells both of CY110 and of *MTL* homozygous strains reported previously by Lan et al. (2002) and Tuch et al. (2010) [20],[21]. Percentages are shown in the brackets.

Similar to the *MTL* homozygous strains, opaque and white cells of the *MTL* **a**/α strain CY110 specialized in their metabolic pathways (Table S4, sheet 2). Fermentative metabolism–associated genes were highly expressed in white cells of CY110 (e.g., glucose transporter genes *HGT6*, *HGT7*, and *HGT8*), while oxidative metabolism–associated genes were highly expressed in opaque cells (e.g., isocitrate dehydrogenase *IDP2*, malate synthase *MLS1*, acyl-CoA oxidase *POX1*, and 3-hydroxyacyl-CoA epimerase genes *FOX2* and *FOX3*). Moreover, the differentially expressed genes in white and opaque cells of CY110, which were also found in their *MTL* homozygous counterparts, included genes associated with the metabolism of other nutrients (such as nitrogen and phosphate), cell wall components, stress response, and transcription factors (Table S4, sheet 2).

In *MTL* homozygous strains, only the opaque cell type is mating-competent [10]. Consistently, it has been demonstrated that mating-related genes *MF*α (α-pheromone) and *STE2* (**a**-pheromone receptor) are highly enriched in opaque cells of WO-1, an *MTL*α/α isolate of *C. albicans* [20]. However, the expression levels of either *MF*α or *MF* **a** were not detectable in white and opaque cells of CY110. The expression levels of their receptors *STE2* and *STE3* in opaque cells of CY110 were very low and similar to that of white cells (Table S4, sheet 4). Additionally, the transcriptional expression of the four genes at the *MTL* loci (**a**1, **a**2, α1 and α2) was all detected. These results served to validate the **a**/α cell identity of CY110 and its mating incompetence.

### Wor1, Rfg1, Brg1, and Efg1 Are Involved in the Regulation of White-Opaque Switching in *C. albicans MTL*a/α Strains

*WOR1* is the master regulator of white-opaque switching in *MTL* homozygotes of *C. albicans* and is extensively up-regulated in opaque cells of both *MTL* homozygotes and heterozygotes (Figure 1D and Table S4) [14]–[16]. Deletion of *WOR1* in an *MTL* **a**/α strain SZ306u, a derivative of SZ306, blocked white to opaque switching on all media tested including Lee's GlcNAc medium in 5% CO_2_ (switching frequency<0.03%) (Figure S4). Under this culture condition, the white-to-opaque switching frequency of the wild-type SZ306u (*WOR1/WOR1*) and the single copy mutant (*WOR1/wor1*) were 4.3±1.0% and 0.5±0.3%, respectively, suggesting that the copy number of *WOR1* could affect its own expression and the white-to-opaque switching frequency. Therefore, Wor1 is also essential for the induction of opaque phenotype in *MTL* **a**/α strains.

**Figure 6:**
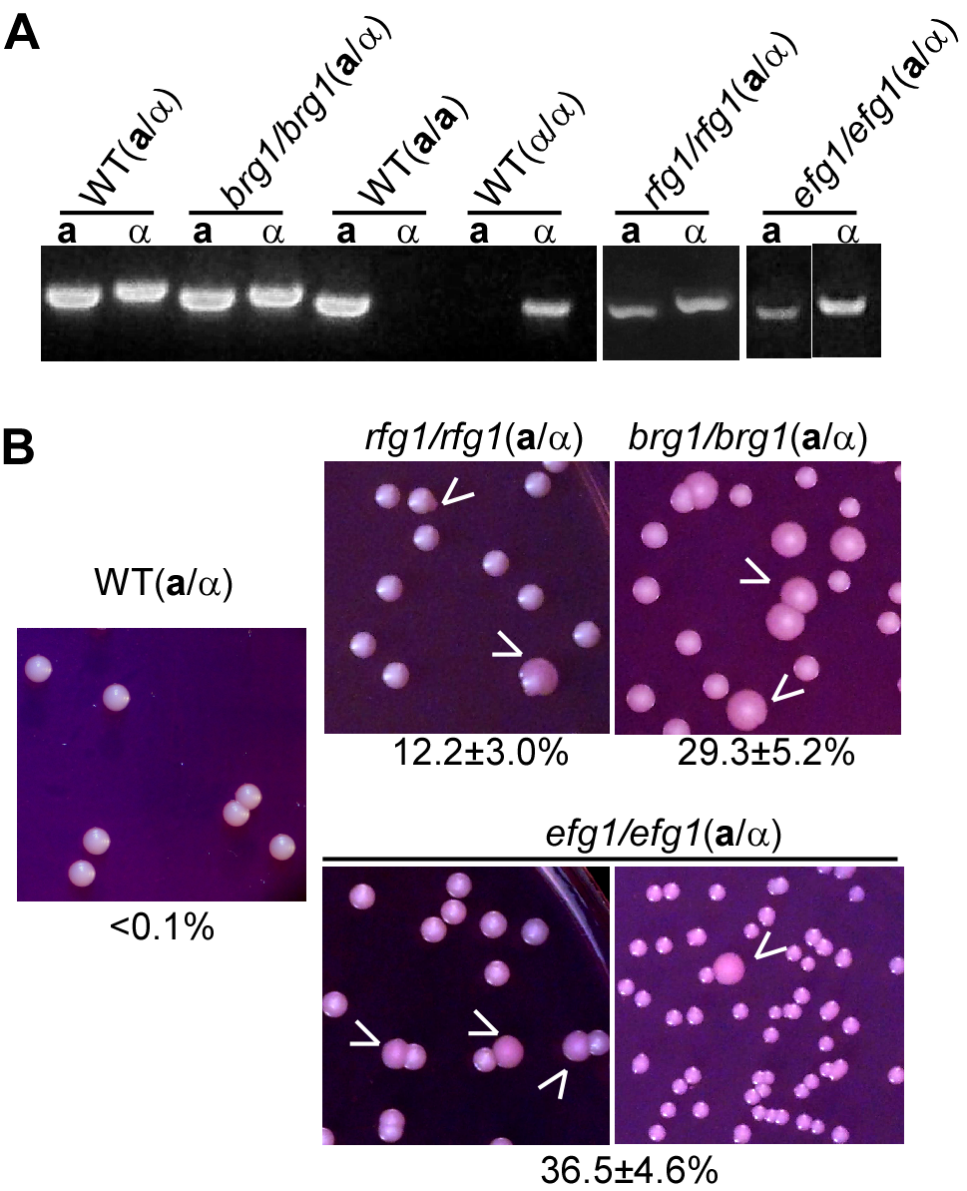
Rfg1, Brg1, and Efg1 regulate white-opaque switching in *MTL*a/α strains. (A) PCR of *MTL* **a**1 and α2 genes of the *rfg1/rfg1*, *brg1/brg1*, and *efg1/efg1* mutants. Primers used for PCR are listed in Table S6. Three characterized WT strains served as *MTL* **a**/α (SN250), α/α (WO-1), and **a**/**a** (GH1012) controls. (B) White-to-opaque switching in *rfg1/rfg1*, *brg1/brg1*, and *efg1/efg1* mutants (*MTL* **a**/α) on Lee's GlcNAc plates. WT (SN250, *MTL* **a**/α), a derivative of SC5314, served as a control. The three mutants were also with the same background of SC5314. Switching frequencies of white-to-opaque are shown below the colony images.

The **a**1/α2 complex inhibits the expression of *WOR1* and thus controls white-to-opaque switching in SC5314 background strains [14]–[16]. The promoter region of *WOR1* is extremely long (>10 kb), indicating the regulation of *WOR1* expression could be very complex. Two facts imply that the **a**1/α2 complex does not work alone to control the expression of *WOR1*. First, even in the *MTL* homozygous strains (that therefore lack the **a**1/α2 complex), the default cell type is the white form, at least in typical laboratory media, indicating some other regulators must repress the expression of *WOR1*. Second, there appears to be only a single binding site of the **a**1/α2 complex on the long promoter region of *WOR1*. To find the regulators coordinately working with the **a**1/α2 complex in repressing *WOR1* expression, we screened a library of ∼160 transcription factor null mutants (of the *MTL* **a**/α genotype) of SC5314 background [22]. The library was suitable for the screening because SC5314 and its derivatives (**a**/α) used for making the mutants are nonswitchable on the Lee's GlcNAc medium. We predicted that inactivating the transcription factors involved in inhibiting *WOR1* expression would lead to the opaque phenotype. And we found three **a**/α mutants (*rfg1/rfg1*, *brg1/brg1*, and *efg1/efg1*) could undergo white-to-opaque switching on the Lee's GlcNAc medium in 5% CO_2_ at 25°C, suggesting the transcription factors Rfg1, Brg1, and Efg1 are involved in the regulation of white-opaque transition in *MTL* **a**/α strains of *C. albicans*. PCR analysis was conducted to confirm that the *MTL* genotype of the *rfg1/rfg1*, *brg1/brg1*, and *efg1/efg1*mutants were **a**/α (Figure 6A). Rfg1 is a member of the HMG domain family of sequence-specific DNA-binding proteins that has been shown to be a regulator of filamentous growth and virulence in *C. albicans* [23],[24]. We observed that the *rfg1/rfg1* mutant (**a**/α) could also form opaque colonies or sectors in Lee's glucose and YPD media when cultured at 25°C for an extended time period (unpublished data). Consistent with the phenotype of *rfg1/rfg1* mutant in white-opaque switching, Northern blots showed that the expression of *RFG1* was enriched in white cells, relative to opaque cells in *C. albicans MTL* **a**/α strains (Figure 1D). Brg1, a GATA-type zinc finger transcription factor, has been characterized as a regulator of filamentous growth, biofilm formation, and virulence [22],[25]. Efg1 is a bHLH domain containing transcription factor required for maintaining the white cell phenotype of *C. albicans MTL* homozygotes [26]. The *efg1/efg1* null mutants of *MTL* **a**/α strains could not switch to opaque in glucose containing medium [27]. However, both *brg1/brg1* and efg1/efg1 mutants of *MTL* **a**/α strains could indeed undergo white-to-opaque switching on Lee's GlcNAc medium (Figure 6B). Our findings indicate that numerous environmental signals converge on Wor1 and regulate the ability of *C. albicans* cells to undergo white-opaque switching.

## Discussion

For decades, white-opaque switching was observed in only a minority (<10%) of natural *C. albicans* isolates: those that were homozygous at the mating-type locus [2],[4]. How does this species maintain such a complex switching system if the majority of strains (which are **a**/α) do not do it? One possibility is that white-opaque switching in *C. albicans* has been maintained as a means to attain mating competency [10]. However, *C. albicans* populations in the host are primarily clonal, indicating that, if parasexual mating actually occurs in nature, its role may not be to generate genetic diversity [28]. In this study, we have generated evidence for a different explanation for the widespread maintenance of white-opaque switching in *C. albicans* clinical isolates. We show that many naturally occurring *MTL* **a**/α strains of *C. albicans* can indeed undergo white-opaque switching, with the opaque phenotype of *MTL* **a**/α strains of *C. albicans* being largely similar to that of *MTL* homozygotes, except that they do not mate. Although such switching of **a**/α strains does not readily occur under typical laboratory conditions, we show that the combination of GlcNAc and CO_2_ are strong inducers of switching in **a**/α strains. Importantly, some **a**/α strains can undergo white-to-opaque switching at 37°C, the physiological temperature of the human host. These conditions are believed to be present in host niches such as the gut, where glucose is limiting and the carbon sources are largely from GI mucus and cell debris of microbes [29]. Together with our recent discovery of white-opaque switching in *MTL* **a**/α heterozygotes of *C. tropicalis* [12], our findings thus generalize white-opaque switching to strains with all mating-type configurations and suggest that the ability to switch is conserved in *C. albicans* and *C. tropicalis*.

We have shown that opaque cells of *MTL* **a**/α isolates of *C. albicans* share many features with opaque cells of *MTL* homozygotes. However, there are some important differences. For example, opaque cells of *MTL* **a**/α isolates undergo mass conversion to white cells on glucose containing media, while opaque cells of *MTL* homozygotes are very stable. Thus, **a**/α opaque cells are not as stable as opaque **a** or α cells and require the continuous presence of the environmental signals. Secondly, opaque cells of *MTL* **a**/α isolates are mating-incompetent.

The MTL**a**1/α2 complex inhibits the expression of the master regulator *WOR1*, thereby blocking white-opaque switching in the laboratory strain SC5314 [14]–[16], which is an **a**/α strain. However, in the **a**/α strains described here, white-opaque switching is permitted; the **a**1/α2 complex “turns it down” but does not completely block white-opaque switching. The long upstream region of *WOR1* implies that multiple environmental signals and transcriptional regulators feed into it, and thus it is easy to imagine that strains could vary in the precise response of Wor1 to environmental signals. We have demonstrated that more than one third of natural isolates of *MTL* **a**/α *C. albicans* strains tested in this study formed opaque or opaque-sectored colonies on Lee's GlcNAc plates in 5% CO_2_. We propose that the white-opaque phenotypic transition itself is a general feature of *C. albicans*, but the quantitative response of the switch to features of the environment and to the mating type configuration differs from strain to strain.

**Figure 7:**
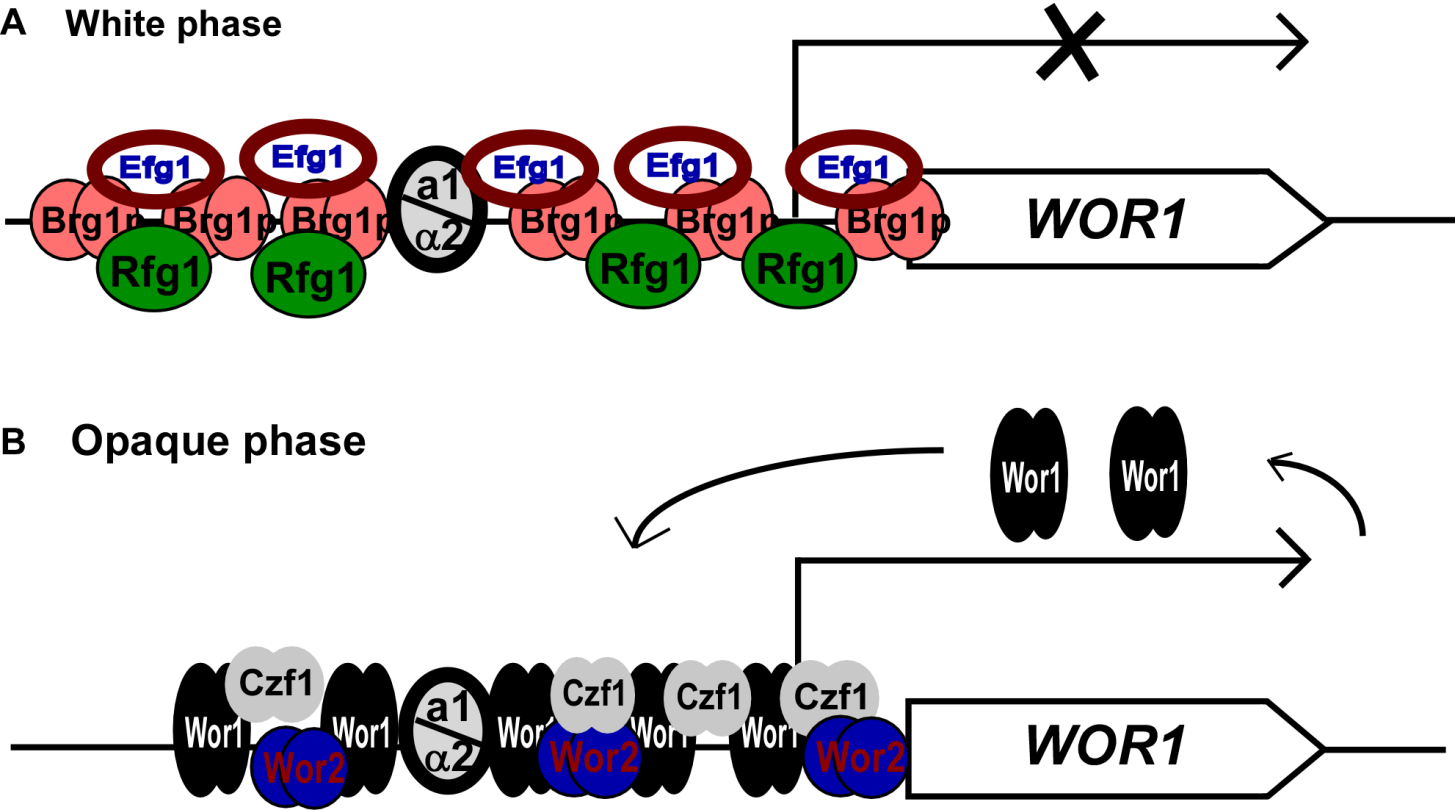
Models of the transcriptional regulation of the master regulator *WOR1* in *MTL*a/α strains of *C. albicans*. (A) In white cells. Negative regulators Brg1, Efg1, and Rfg1, together with the MTL**a**1/α2 complex, coordinately repress the transcription of *WOR1*. (B) In opaque cells. Positive regulators Wor2 and Czf1, together with Wor1, coordinately regulate the transcription of *WOR1* via a positive feedback loop.

The regulation of white-opaque switching in *MTL* homozygotes involves an interlocking transcriptional circuit, in which Wor1 occupies the central position [27]. We propose that Wor1 also acts as a master regulator in the process of white-opaque switching in *MTL* heterozygotes of *C. albicans*. Ectopic expression of *WOR1* in the “non-switchable” *MTL* **a**/α strain CAI4, a derivative of SC5314, induces white-to-opaque switching on glucose-containing laboratory media, suggesting that Wor1, if ectopically expressed, can override the repressing effect of the **a**1/α2 complex on the white-to-opaque transition [14],[16]. By screening a deletion mutant library of *C. albicans*, we have identified three transcription factors, Rfg1, Brg1, and Efg1, involved in the regulation of white-opaque switching in *MTL* **a**/α strains. These three transcription factors inhibit opaque cell formation in *MTL* **a**/α strains since their null mutants are capable of switching between white and opaque cell types. Consistent with the phenotype of their null mutants, the transcriptional expression of *RFG1* and *EFG1* was enriched in white cells of *MTL* **a**/α strains (Figure 1). Nobile et al. have recently demonstrated that Brg1 and Efg1 bind to nearly the entire 10 kb intergenic region between *WOR1* and its adjacent divergent gene *ORF19.4883* [25]. However, the location of the peaks of Brg1 and Efg1 binding were distant from the putative **a**1/α2 cis-regulatory sequence. These results not only provide direct evidence of Brg1 and Efg1 binding to the promoter of *WOR1*, but also indicate that they may work together with the **a**1/α2 complex to reduce the expression of Wor1 in white cells and prevent switching to opaque cells. The transcriptional repressor Rfg1 may work in a similar manner as Efg1 and Brg1. We propose that inactivation of any of these three regulators would lead to increased expression of *WOR1*, which then initiates a self-positive feedback loop to induce the opaque cell phenotype (Figure 7A). Together with Wor1, additional transcriptional regulators, such as the positive regulators Wor2 and Czf1, coordinately regulate the expression of *WOR1* by binding directly to the *WOR1* upstream intergenic region (A.D.H., C.J.N., and A.D.J. unpublished data), and maintain the cells in the opaque phase (Figure 7B). Consistent with the model in Figure 7B, deletion of *WOR2* or *CZF1* results in increased opaque-to-white switching frequencies [27].

In summary, we have shown that, in many naturally occurring *C. albicans* strains, the **a**1/α2 repressor is not an absolute block to white-opaque switching as it is in the standard laboratory strain SC5314. Rather, the **a**1/α2 activity reduces switching frequency (and renders the opaque form less stable) in these newly described strains, but this reduction can be partially overcome by the addition of GlcNAc and CO_2_ to the growth medium. We propose that the **a**1/α2 repressor and other regulators (including Efg1, Brg1, and Rfg1) as well as these environmental signals all impinge on the long regulatory region of Wor1, the master regulator of white-opaque switching. This information is somehow integrated by the Wor1 regulatory region, and the level of Wor1 transcription is set accordingly. Because Wor1 appears to be the major determinant of the white-opaque switch frequency [30], the model can account for nearly all the observations in this article.

The most important implication of the work is that all strains of *C. albicans* (not just strains that are homozygous at the mating type locus, as previously believed) can undergo white-opaque switching if the appropriate signals are present in the growth medium. Thus, we propose that multiple environmental inputs combined with internal transcriptional regulators can activate white-opaque switching in virtually all *C. albicans* strains. White-opaque switching, in essence, produces two radically different types of cells from the same genome, thereby explaining the ability of *C. albicans* to occupy different niches in the host. We believe that the discovery of white-opaque switching in naturally occurring **a**/α strains accounts for the widespread conservation of the white-opaque switching machinery.

## Materials and Methods

### Culture Conditions, Strains, and Plasmids

The strains used in this study are listed in Table S5. YPD (20 g/L glucose, 20 g/L peptone. 10 g/L yeast extract) was used for routine growth. Lee's + glucose and Lee's + GlcNAc media were used for mating and white-opaque switching assays [17].

The plasmid pSFS2A-URA3 was generated by inserting two DNA fragments containing sequences homologous to the 5′- and 3-terminals of *C. albicans URA3* gene into the *Apa*I/*Xho*I and *Sac*II/*Sac*I sites of pSFS2A [31]. The auxotrophic strain SZ306u for uridine was constructed by disruption of one copy of *URA3* with the linearized plasmid pSFS2A-URA3 and then grown on 5-fluoroorotic acid (5-FOA) containing medium. The white-opaque switching-competence of SZ306u was then confirmed. SZ306 and RVVC10 were converted to SZ306a and RVVC10α by deletion of one *MTL* allele with the plasmid T2A-MTL (Srikantha and Soll, unpublished). The first copy of *WOR1* was deleted with the PCR product of pGEM-URA3 with the primers of WOR1-5DR and WOR1-3DR in SZ306u [32]. The second copy of *WOR1* was then deleted with the linearized plasmid T2A-WOR1 [33]. A couple of primer sets were used to confirm the correct disruption of *WOR1* in SZ306u.

To construct the *WOR1/WOR1::WOR1p-GFP*, *EFG1/EFG1::EFG1p-GFP*, and *WH11/WH11::WH11p-GFP* strains, CY110 was transformed with PCR products of the GFP-caSAT1 fragment (amplified from the template plasmid pNIM1 with GFP reporter primers, Table S6) [34]. The forward primers contained 60 bp of hanging homology to the promoter region of *WOR1*, *WH11*, or *EFG1*, while the reverse primers contained 60 bp of hanging homology to the 3′-UTR of *WOR1*, *WH11*, or *EFG1*. Correct integration of the transformations was verified by genomic DNA PCR with checking primers. All primers used in this study are listed in Table S6.

### Microsatellite CAI Genotyping

The CAI genotypes of *C. albicans* isolates were determined as described by Sampaio et al. (2003) [35]. Briefly, the microsatellite locus CAI was amplified by PCR using a pair of primers (forward, 5′- ATG CCA TTG AGT GGA ATT GG -3′; reverse, 5′- AGT GGC TTG TGT TGG GTT TT -3′). The forward primer was 5′ fluorescently labeled with 6-carboxyfluorescein. The sizes of the amplicons were determined by GeneScan analysis using a DNA sequencer, and the number of trinucleotide repeat units in each fragment was calculated. Because of the diploid nature of *C. albicans*, the CAI genotype of a strain is determined by the repeat number in both alleles of the locus. For example, a strain with a genotype CAI 17–21 means that one allele of the locus contains 17 trinucleotide repeats and the other 21.

### White-Opaque Switching and Mating Assays

White-opaque switching and mating assays were performed as previously described [36]. The cells were incubated in air or in 5% CO_2_ for 4 to 10 days as indicated in the main text. We examined 350 to 500 colonies for each strain. More were tested for nonswitchable strains or on nonconducive media. To verify the colony phenotype, several randomly selected colonies were examined for the cellular morphology. The dye phloxine B, which exclusively stains opaque colonies red, was added to the media. Scanning electron microscopy (SEM) assay was described as we described previously [22]. To observe the mating response, 10^6^ cells of each of the two mating strains indicated in the text were mixed and spotted onto Lee's GlcNAc agar and incubated at 25°C for 4 days. At least 1×10^7^ cells of each mating patch were examined with a light microscopy. Quantitative mating assay was performed as previously described with slight modifications [10]. Briefly, the mating experiments were performed on Lee's GlcNAc medium at 25°C. The experimental opaque cell samples were collected from Lee's GlcNAc medium plates. To test the mating ability of the *MTL* **a**/α strain (SZ306u), 1×10^6^ of *MTL* **a**/**a** (or *MTL*α/α) cells and 1×10^6^ of *MTL* **a**/α cells were mixed and cultured on Lee's GlcNAc medium plates for 48 hours. The mating mixtures were resuspended, diluted, and plated onto three types of selectable plates (without uridine, or arginine, or both) for prototrophic growth. Mating efficiencies were calculated as previously described [14].

The library of transcription factor mutants contains the TF mutants generated by the Johnson lab [37] and strains collected from Candida community [36]. Cells of each mutant were plated onto Lee's GlcNAc plates and incubated at 25°C for 7 to 10 days. Opaque colonies were replated and tested for the *MTL* genotype with PCR.

### RNA Extraction, Northern Blot Analysis, and RNA-Seq

White and opaque cells were cultured on Lee's glucose and Lee's GlcNAc plates at 25°C for 4 days, and then inoculated in Lee's glucose and Lee's GlcNAc liquid media, respectively. Cells were collected from the cultures in exponential phase for RNA extraction. Purified PCR products of *WH11*, *OP4*, *EFG1*, *WOR1*, and *RFG1* genes were used to make probes for Northern blot hybridization. Primers used for the PCR reactions are listed in Table S6. RNA-Seq analysis was performed by the company BGI-Shenzhen.

### Animal Infections

Systemic infection of mice was performed elsewhere [22]. Male ICR mice (18–22 g) were used in this study. Each male mouse was intravenously injected with 200 µl 1× PBS containing 2×10^6^ cells via the tail vein. Three to four mice per strain were used for the injections. Mice were sacrificed on the 3^rd^ day postinfection. The fungal burdens of kidneys and livers were tested. The systemic infection experiments were performed for four independent times. Newborn ICR mice (2 to 4 days) were used for cutaneous infection. The experiments were performed according the protocol reported by Kvaal et al. [8]. The skin colonization by *C. albicans* cells was assessed by scanning electron microscopy. The skin infection experiments were performed for three independent trials. All animal experiments were performed according to the guidelines approved by the Animal Care and Use Committee of the Institute of Microbiology, Chinese Academy of Sciences. The present study was approved by the Committee.

### Accession Number

The RNA-Seq data have been deposited into the NCBI Gene Expression Omnibus (GEO) portal under the accession number GSE43938.

## Supporting Information

Figure S1 White-opaque switching in six natural *MTL*a/α strains of *C. albicans*. Cells were first patched on YPD plates and incubated at 37°C for 2 days. Then, the cells were replated onto Lee's GlcNAc plates and incubated at 25°C in 5% CO_2_ for 6 days. Partial opaque colonies were indicated with white arrows.
(JPG)

Figure S2 Opaque-to-white switching in SZ306 (a/α) and its derivative, SZ306a (a/Δ). Opaque cells from Lee's GlcNAc plates were plated and incubated under four conditions indicated at 25°C. Lee's glucose or GlcNAc medium was used for cell growth. Switching frequencies are shown below the images.
(JPG)

Figure S3 Opaque cells of *MTL*a/α strains of *C. albicans* are stable in Lee's GlcNAc medium at 37°C. Opaque cells of three natural *MTL*a/α strains were plated onto Lee's glucose or GlcNAc plates and incubated at 37°C for 3 days. The cellular morphology of a representative colony is shown.
(JPG)

Figure S4 Deletion of *WOR1* blocks GlcNAc and CO_2_ induced white-to-opaque switching in *MTL* heterozygotes of *C. albicans*. White cells were plated onto Lee's GlcNAc plates and incubated in 5% CO_2_ for 5 days at 25°C. White arrows indicated opaque colonies. Switching frequencies (Swit. freq.) are shown below the images.
(JPG)

Table S1 White-opaque switching in natural strains isolated in China.
(XLS)

Table S2 White-opaque switching in natural strains of five different genetic clades.
(XLS)

Table S3 Opaque-to-white switching in a/α, a/Δ, and Δ/α strains.
(DOC)

Table S4 RNA-Seq analysis of white and opaque cells of CY110.
(XLS)

Table S5 Strains used in this study.
(DOC)

Table S6 Primers used in this study.
(DOC)

## Abbreviations

CFU: colony-forming units
GI tract: gastrointestinal tract
GlcNAc: N-acetylglucosamine
MTL: mating type-like
ORF: open reading frame
SEM: scanning electron microscopy
WT: wild type
