# Supplementary figures and images for: White-Opaque Switching in Natural MTLa/α Isolates of Candida albicans: Evolutionary Implications for Roles in Host Adaptation, Pathogenesis, and Sex
Source: PLoS Biol. 2013 Mar 26;11(3):e1001525. doi: 10.1371/journal.pbio.1001525 (PMC3608550; doi:10.1371/journal.pbio.1001525)

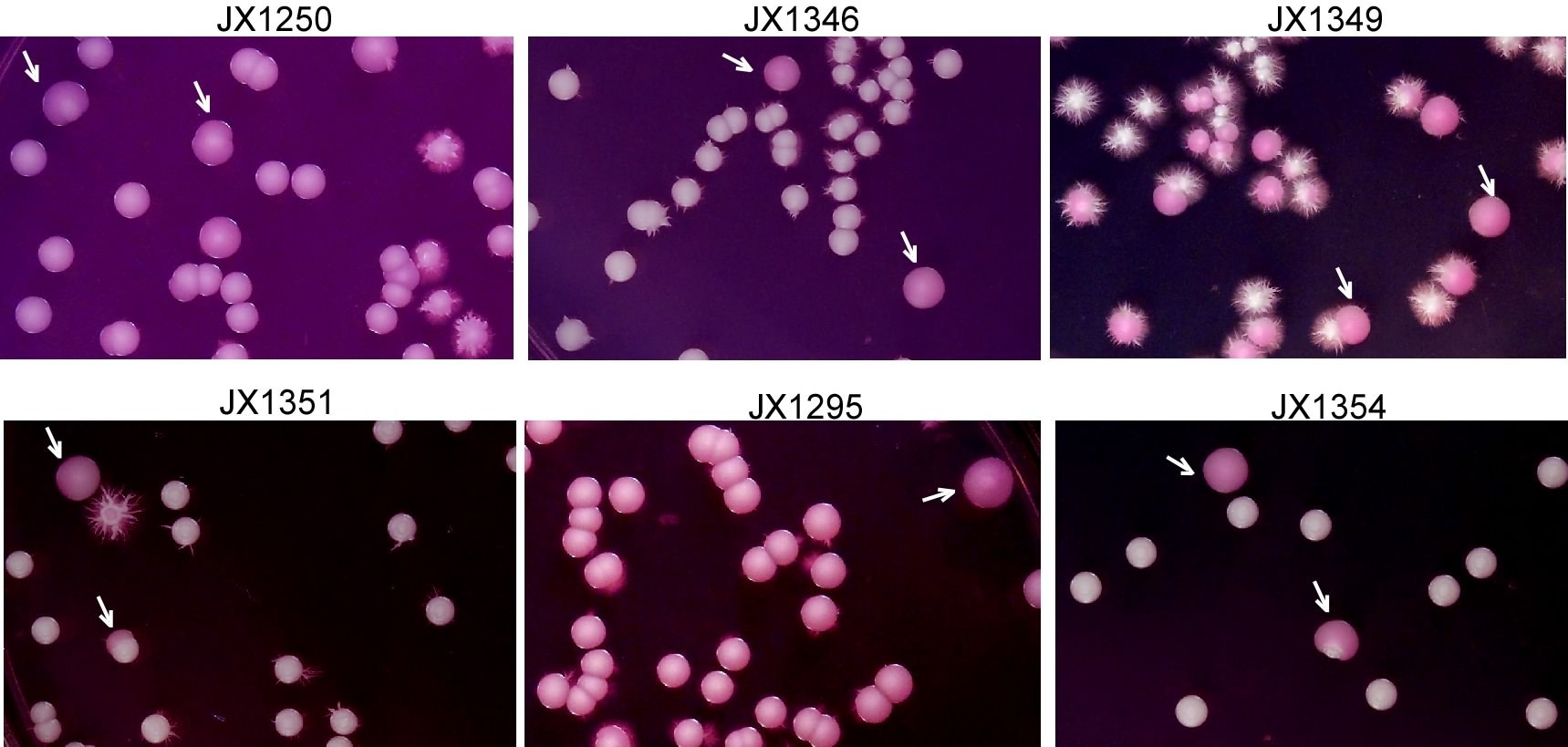

Supplement: Figure S1 — White-opaque switching in six natural MTLa/α strains of C. albicans. Cells were first patched on YPD plates and incubated at 37°C for 2 days. Then, the cells were replated onto Lee's GlcNAc plates and incubated at 25°C in 5% CO2 for 6 days. Partial opaque colonies were indicated with white arrows. (JPG) [file pbio.1001525.s001.jpg]

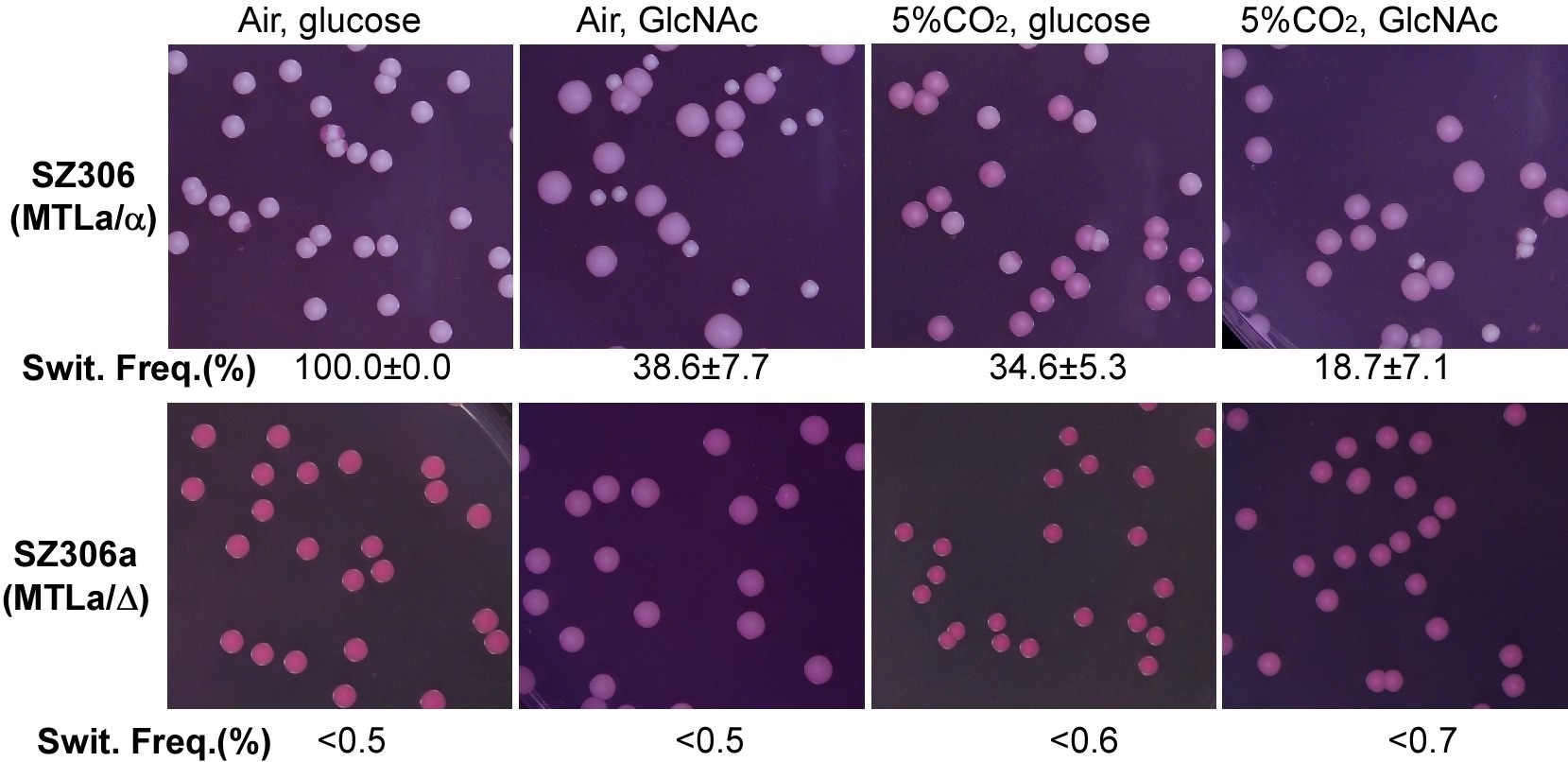

Supplement: Figure S2 — Opaque-to-white switching in SZ306 (a/α) and its derivative, SZ306a (a/Δ). Opaque cells from Lee's GlcNAc plates were plated and incubated under four conditions indicated at 25°C. Lee's glucose or GlcNAc medium was used for cell growth. Switching frequencies are shown below the images. (JPG) [file pbio.1001525.s002.jpg]

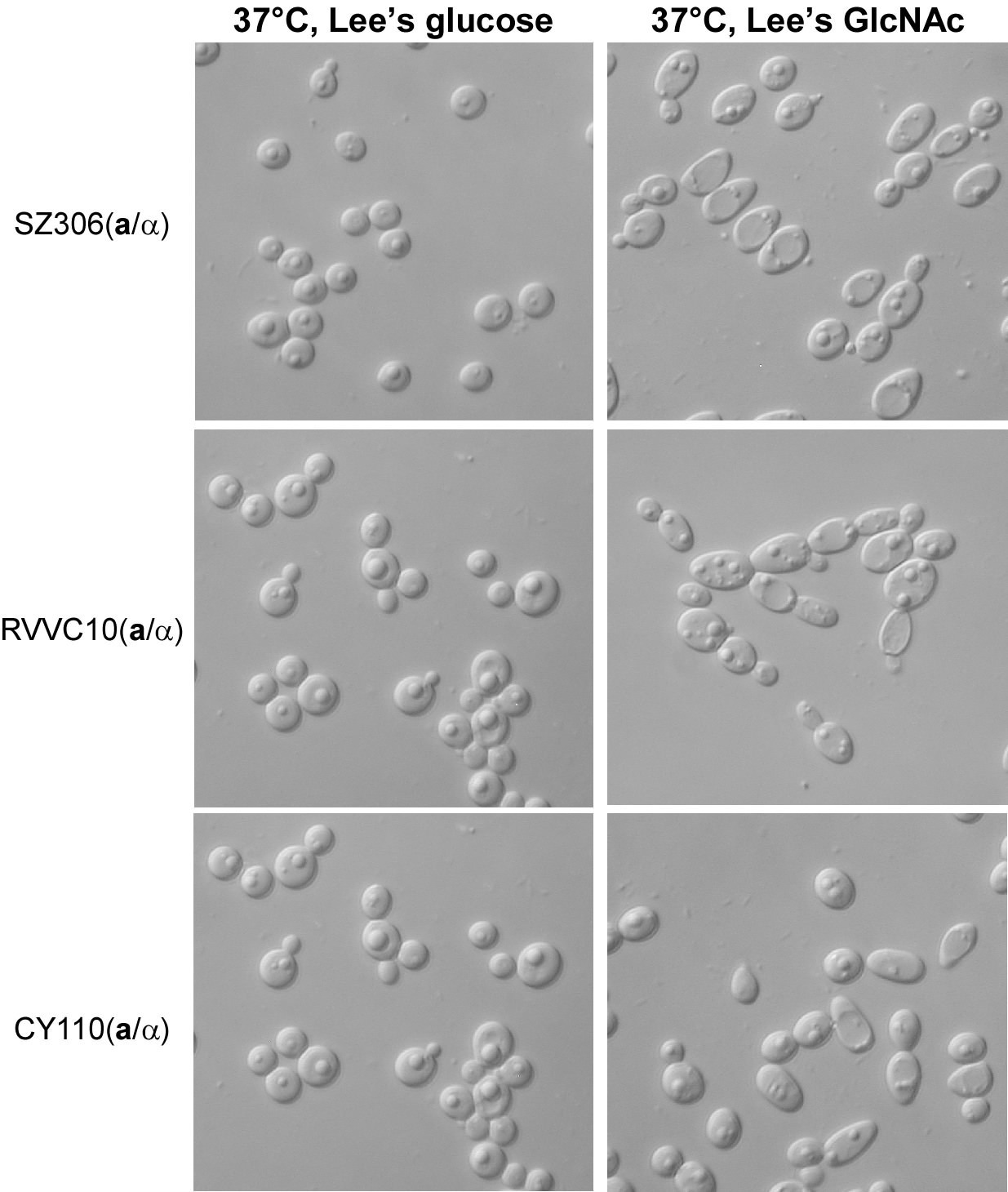

Supplement: Figure S3 — Opaque cells of MTLa/α strains of C. albicans are stable in Lee's GlcNAc medium at 37°C. Opaque cells of three natural MTLa/α strains were plated onto Lee's glucose or GlcNAc plates and incubated at 37°C for 3 days. The cellular morphology of a representative colony is shown. (JPG) [file pbio.1001525.s003.jpg]

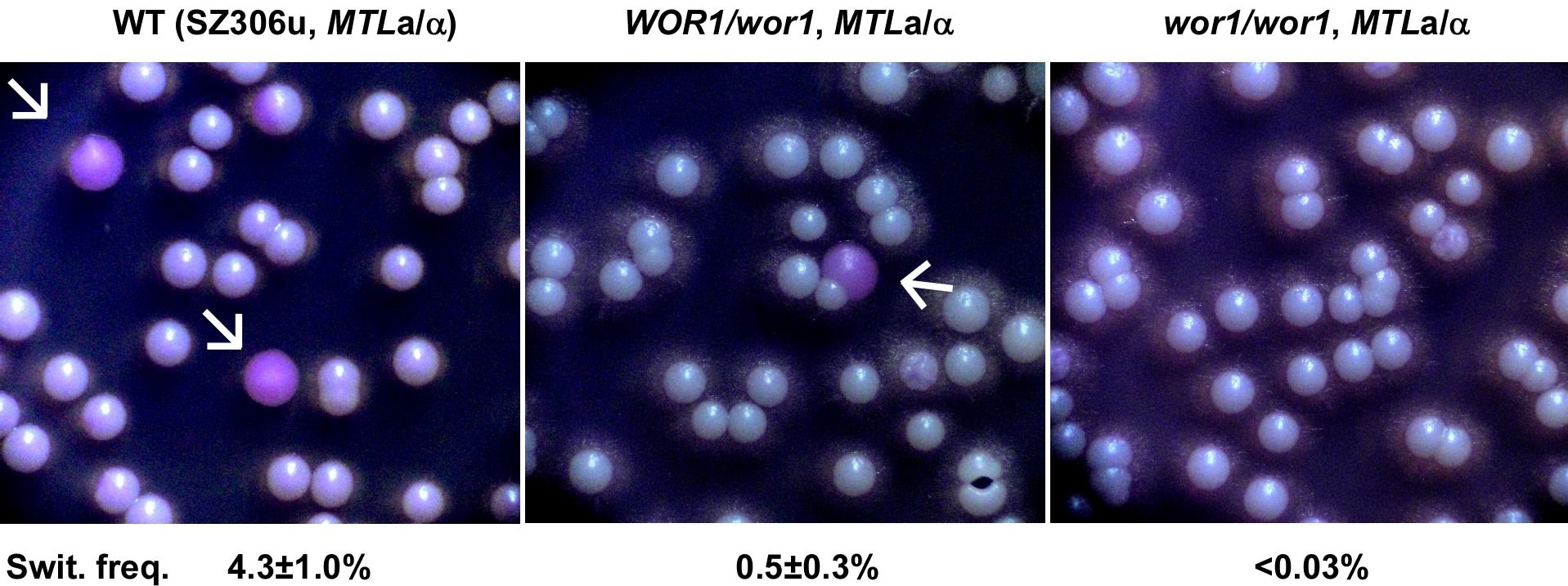

Supplement: Figure S4 — Deletion of WOR1 blocks GlcNAc and CO2 induced white-to-opaque switching in MTL heterozygotes of C. albicans. White cells were plated onto Lee's GlcNAc plates and incubated in 5% CO2 for 5 days at 25°C. White arrows indicated opaque colonies. Switching frequencies (Swit. freq.) are shown below the images. (JPG) [file pbio.1001525.s004.jpg]
