# Supplementary material for: White-Opaque Switching in Natural MTLa/α Isolates of Candida albicans: Evolutionary Implications for Roles in Host Adaptation, Pathogenesis, and Sex
Source: PLoS Biol. 2013 Mar 26;11(3):e1001525. doi: 10.1371/journal.pbio.1001525 (PMC3608550; doi:10.1371/journal.pbio.1001525)
Supplement: Table S3 — Opaque-to-white switching in a/α, a/Δ, and Δ/α strains. (DOC) [file pbio.1001525.s007.doc]

**Table S3. Opaque-to-white switching in** a**/α,** a**/- and** -**/α strains**

| **Strain** | **Culture condition** | **Switching frequency (%)** |
| --- | --- | --- |
| **SZ306 (**a**/α)** | Glucose, air | 100.0±0.0 |
|  | GlcNAc, air | 38.6±7.7 |
|  | Glucose, 5%CO_2_ | 34.6±5.3 |
|  | GlcNAc, 5%CO_2_ | 18.7±7.1 |
| **SZ306a (**a**/-)** | Glucose, air | <0.5 |
|  | GlcNAc, air | <0.5 |
|  | Glucose, 5%CO_2_ | <0.6 |
|  | GlcNAc, 5%CO_2_ | <0.7 |
| **RVVC10 (**a**/α)** | Glucose, air | 100.0±0.0 |
|  | GlcNAc, air | 41.2±7.0 |
|  | Glucose, 5%CO_2_ | 53.3±7.7 |
|  | GlcNAc, 5%CO_2_ | 11.4±2.2 |
| **RVVC10α (-/α)** | Glucose, air | <0.8 |
|  | GlcNAc, air | <0.7 |
|  | Glucose, 5%CO_2_ | <0.8 |
|  | GlcNAc, 5%CO_2_ | <0.7 |

Opaque cells from Lee’s GlcNAc plates in 5% CO_2_ were re-plated onto Lee’s glucose or GlcNAc plates and grew for 5 days in air or 5% CO_2_ at 25°C. The carbon source used was indicated in the table. Switching frequency represented the percentage of white or colonies containing white cell sectors.
