# Supplementary material for: White-Opaque Switching in Natural MTLa/α Isolates of Candida albicans: Evolutionary Implications for Roles in Host Adaptation, Pathogenesis, and Sex
Source: PLoS Biol. 2013 Mar 26;11(3):e1001525. doi: 10.1371/journal.pbio.1001525 (PMC3608550; doi:10.1371/journal.pbio.1001525)
Supplement: Table S5 — Strains used in this study. (DOC) [file pbio.1001525.s009.doc]

**Table S5. Strains used in this study**

| **Strain** | **Parent strain** | **Genotype** | **Reference** |
| --- | --- | --- | --- |
| SZ306 |  | Clinical isolate, *MTL***a***/α* | [1] |
| RVVC10 |  | Clinical isolate, *MTL***a***/α* | This study |
| CY110 |  | Clinical isolate, *MTL***a***/α* | [2] |
| SZ306a | SZ306 | Clinical isolate, *MTL***a***/mtlα::SAT1* | This study |
| RVVC10α | RVVC10 | Clinical isolate, *mtl***a***::SAT1/MTLα* | This study |
| SZ306u | SZ306 | As SZ306, but *ura3::FLP/ura3::FLP* | This study |
| *wor1/wor1* | SZ306u | As SZ306, but *wor1::FLP-SAT1-FLP/wor1::URA3* | This study |
| SN250 | CAI4 | *MTL***a***/α ura3::imm434::URA3-IRO1/ura3::imm434 arg4::hisG/arg4::hisG his1::hisG/his1::hisG leu2::hisG::CdHIS1/leu2::hisG::CmLEU2* | [3] |
| *brg1/brg1* | SN152 | *MTL***a***/α ura3::imm434::URA3-IRO1/ura3::imm434 arg4::hisG/arg4::hisG his1::hisG/his1::hisG leu2::hisG /leu2::hisG brg1::CdHIS1/brg1::CmLEU2* | [3] |
| HLC52 | CAI4 | *MTL****a****/α efg1::hisG/efg1::hisG-URA3-hisG ura3::imm434 /ura3::1 imm434* | [4] |
| CaWY5 | BWP17 | *MTL****a****/α rfg1::HIS1/rfg1::URA3* | [5] |
| WUM5A | WO-1 | *MTLα/α ura3-1::FRT/ura3-2::FRT* | [6] |
| GH1013 | BWP17 | *MTL****a/a*** *ura3::imm434/ura3::imm434 his1::hisG/his1::hisG arg4::hisG/arg4::hisG* | [7] |
| WO-1 |  | *MTLα/α* | [8] |
| GH1012 | CAI4 | *MTL***a**/**a** *ura3::imm434/ura3::imm434* | [7] |
| *rfg1/rfg1* | SN152 | *MTL***a***/α ura3::imm434::URA3-IRO1/ura3::imm434 arg4::hisG/arg4::hisG his1::hisG/his1::hisG leu2::hisG /leu2::hisG rfg1::CdHIS1/rfg1::ARG4* | This study |
| WOR1p-GFP |  | As CY110, but *WOR1/WOR1::WOR1p-GFP* | This study |
| WH11p-GFP |  | As CY110, but *WH11/WH11::WH11p-GFP* | This study |
| EFG1p-GFP |  | As CY110, but *EFG1/EFG1::EFG1p-GFP* | This study |
| SN152a | SN152 | *MTL***a**/αΔ*::SAT1 ura3::imm434::URA3-IRO1/ura3::imm434 arg4::hisG/arg4::hisG his1::hisG/his1::hisG leu2::hisG /leu2::hisG* | This study |

**References：**

1. Li, J., Fan, S.R., Liu, X.P., Li, D.M., Nie, Z.H., Li, F., Lin, H., Huang, W.M., Zong, L.L., Jin, J.G., et al. (2008). Biased genotype distributions of Candida albicans strains associated with vulvovaginal candidosis and candidal balanoposthitis in China. Clinical infectious diseases : an official publication of the Infectious Diseases Society of America *47*, 1119-1125.

2. Ge, S.H., Xie, J., Xu, J., Li, J., Li, D.M., Zong, L.L., Zheng, Y.C., and Bai, F.Y. (2012). Prevalence of specific and phylogenetically closely related genotypes in the population of Candida albicans associated with genital candidiasis in China. Fungal genetics and biology : FG & B *49*, 86-93.

3. Homann, O.R., Dea, J., Noble, S.M., and Johnson, A.D. (2009). A phenotypic profile of the Candida albicans regulatory network. PLoS Genet *5*, e1000783.

4. Lo, H.J., Kohler, J.R., DiDomenico, B., Loebenberg, D., Cacciapuoti, A., and Fink, G.R. (1997). Nonfilamentous C. albicans mutants are avirulent. Cell *90*, 939-949.

5. Hu, C.J., Bai, C., Zheng, X.D., Wang, Y.M., and Wang, Y. (2002). Characterization and functional analysis of the siderophore-iron transporter CaArn1p in Candida albicans. The Journal of biological chemistry *277*, 30598-30605.

6. Strauss, A., Michel, S., and Morschhauser, J. (2001). Analysis of phase-specific gene expression at the single-cell level in the white-opaque switching system of Candida albicans. J Bacteriol *183*, 3761-3769.

7. Huang, G., Srikantha, T., Sahni, N., Yi, S., and Soll, D.R. (2009). CO(2) regulates white-to-opaque switching in Candida albicans. Curr Biol *19*, 330-334.

8. Slutsky, B., Staebell, M., Anderson, J., Risen, L., Pfaller, M., and Soll, D.R. (1987). "White-opaque transition": a second high-frequency switching system in Candida albicans. J Bacteriol *169*, 189-197.
