# Supplementary material for: White-Opaque Switching in Natural MTLa/α Isolates of Candida albicans: Evolutionary Implications for Roles in Host Adaptation, Pathogenesis, and Sex
Source: PLoS Biol. 2013 Mar 26;11(3):e1001525. doi: 10.1371/journal.pbio.1001525 (PMC3608550; doi:10.1371/journal.pbio.1001525)
Supplement: Table S6 — Primers used in this study. (DOC) [file pbio.1001525.s010.doc]

**Table S6. Primers used in this study**

| **Name** | **Sequence (5’ to 3’)** | **Purpose** |
| --- | --- | --- |
| probeEFG1F | CGAGTGATTCAAACCGGAGT | Northern probes |
| probeEFG1R | CTGAACTTGGGGTGATTGGT |
| probeWH11F | CCGACTTAGGTAGAAAAGATATTGG |
| probeWH11R | TTGGAGTCACCAAAAATAGCA |
| probeOP4F | CTCGGTGGCAGCTCTAGTTC |
| probeOP4R | CAGCTTGACCAGCAGCATTA |
| probeWOR1F | AGTGGTGGTAATGGCAATGG |
| probeWOR1R | CGACCCAGAAGAATTTCCAA |
| probeRFG1F | AAGCTCCACACTTATCAATC |
| probeRFG1R | AATGCATTTCTTGGTCGAG |
| MTLaF | TTGAAGCGTGAGAGGCTAGGAG | PCR of *MTL***a**1 or alpha2 |
| MTLaF | ATCAATTCCCTTTCTCTTCGATTAGG |
| MTLa1phaF | TTCGAGTACATTCTGGTCGCG |
| MTLalphaF | TGTAAACATCCTCAATTGTACCCGA |
| Wor-5DR | GTACACTGACATCTCAAACATCAAAGATACACTTATTTCAAGTTCAATAGTGAAGTTTCAgttttcccagtcacgacgtt | Deletion of *WOR1* |
| Wor-3DR | AACAATCTTACCAACAGTAAGTATAATCATCTAAACCTATAGTACAACACAACATACACCtgtggaattgtgagcggata |
| WOR1-5DF | TATTGCAGCAACAGATTTCCAC | *WOR1* deletion confirmation  (first copy) |
| WOR1-5DR | TTCCTTAGTGGTATCAACGTC |
| WOR1-5DF | CAATCAAAGGTGGTCCTTCTAG |
| WOR1-5DR | CAAGGCGTCATCATATCATTC |
| Wor1CHF | CAACAACAACAACAACAACACC | *WOR1* deletion confirmation  (second copy) |
| Wor1CHR | TGCCATTACCACCACTAACAC |
| pSFR | CAATGAAATCCAGACAGTCGAG |
| pSFF | CGATTAGAGACACAAACGAAC |
| WH11pGFP-F | ATATTTCCCTTCCTTTTTTTTTATCATTATAAAACAAAACAACAAACAGAACAATTAAACAATATAAATAGTCGACAAAG | WH11p-GFP reporter |
| WH11pGFP- R | ATCTAAGCGAAACAAAATCATAGCAAGACTTCAACTCAATTGAGGTTACTCACTCATTGTgaccacctttgattgtaaatag |
| WOR1pGFP-F | GGGGGTTGAAAATTTTAAACTGAAAACAACAATTAGTATAATTCAATTCAATATTAAGCAAATATAAATAGTCGACAAAG | WOR1p-GFP reporter |
| WOR1pGFP-R | TGTAAAAAAAAACACCTGAATGAGCCCCAAAATAATAACAGAATCACCGTATTAATTCAAgaccacctttgattgtaaatag |
| EFG1pGFP-F | TTAACCCATTAACGAATTAAGATTTGTTCTATTTGACTACCAAGAATATAACCCATATTAAATATAAATAGTCGACAAAG | EFG1p-GFP reporter |
| EFG1pGFP-R | CAGTGCTAGCTGATTGATTAGCTTGATGTTGTTGGGGTGAAGGGTGAACTGAACCTTGAGgaccacctttgattgtaaatag |
| WH11pGFP-DF | AGAACTACTTGCTATCCTGC | GFP reporter checking |
| WOR1pGFP-DF | GAAGACCATTTACGCCGCAC |
| EFG1pGFP-DF | ACATTAGTTGCTCAGGTCAC |
| GFP-DR | AGCATTGAAGACCATACGCG |
